# Graphitic Carbon Nitride for Photocatalytic Hydrogen Production from Water Splitting: Nano-Morphological Control and Electronic Band Tailoring

**DOI:** 10.3390/nano15010045

**Published:** 2024-12-30

**Authors:** Yongbo Fan, Xinye Chang, Weijia Wang, Huiqing Fan

**Affiliations:** 1 Department of Applied Physics, The Hong Kong Polytechnic University, Hung Hom, Hong Kong 100872, China; yongbfan@polyu.edu.hk; 2 State Key Laboratory of Solidifcation Processing, School of Materials Science and Engineering, Northwestern Polytechnical University, Xi’an 710072, China; yibingchao666@mail.nwpu.edu.cn (X.C.); weijia.wang@nwpu.edu.cn (W.W.)

**Keywords:** graphitic carbon nitride, photocatalysis, hydrogen evolution, morphological control, electronic band tailoring

## Abstract

Semiconductor polymeric graphitic carbon nitride (g-C_3_N_4_) photocatalysts have garnered significant and rapidly increasing interest in the realm of visible light-driven hydrogen evolution reactions. This interest stems from their straightforward synthesis, ease of functionalization, appealing electronic band structure, high physicochemical and thermal stability, and robust photocatalytic activity. This review starts with the basic principle of photocatalysis and the development history, synthetic strategy, and structural properties of g-C_3_N_4_ materials, followed by the rational design and engineering of g-C_3_N_4_ from the perspectives of nano-morphological control and electronic band tailoring. Some representative results, including experimental and theoretical calculations, are listed to show the advantages of optimizing the above two characteristics for performance improvement in photocatalytic hydrogen evolution from water splitting. The existing opportunities and challenges of g-C_3_N_4_ photocatalysts are outlined to illuminate the developmental trajectory of this field. This paper provides guidance for the preparation of g-C_3_N_4_ and to better understand the current state of the art for future research directions.

## 1. Introduction

The development of modern society heavily relies on fossil fuels as traditional energy sources. However, due to the limited availability of fossil fuels, their rapid consumption has led to a serious energy crisis. In addition, the consumption of fossil fuels will inevitably produce harmful emissions, leading to serious environmental problems. Therefore, it is necessary to seek clean and sustainable energy to improve the development of human society [1]. The utilization of solar energy is an important solution because it is a clean, economical, and inexhaustible source of energy. Semiconductor-based photocatalytic technology has gained widespread attention due to its diverse application potential in energy and environmental aspects [2,3,4]. In 1972, Fujishima and Honda first used TiO_2_ photoelectrodes to catalyze the decomposition of water under ultraviolet light irradiation [5]. In 1976, Carey et al. [6] reported the photocatalytic degradation of organic pollutants by TiO_2_ in aqueous solution. In 1979, Inoue and his colleagues studied the photocatalytic reduction of carbon dioxide to various organic compounds using various semiconductor powders such as TiO_2_, ZnO, GaP, SiC, CdS, etc. [7]. Subsequently, extensive research has been conducted on the manufacturing and utilization of high-efficiency semiconductor-based photocatalysts. So far, the development of high-quality semiconductor photocatalysts has become a hot research field in addressing energy shortages and environmental threats.

Currently, hydrogen energy is gradually becoming one of the important carriers of global energy transformation and development [8]. This is partly due to the fact that, excluding nuclear fuel, hydrogen boasts the highest calorific value of any fuel, whether fossil, chemical, or biofuel. It stands at 1.43 × 10^8^ J/Kg, which is three times the energy content of gasoline. On the other hand, hydrogen only reacts with oxygen to generate water during combustion and does not produce carbon emissions, which has profound implications for improving the climate and environment. Fossil fuel hydrogen production and industrial by-product hydrogen occupy the dominant position in the hydrogen production structure with lower costs, but this undoubtedly goes against the concept of sustainable development. Therefore, the concept of “green hydrogen” emerged, which refers to hydrogen gas obtained by decomposing water using renewable energy sources. Solar energy is the largest energy source that can be developed in the world today, and using solar energy to produce hydrogen is considered the most promising and practical method of hydrogen production. In recent years, researchers have extensively studied the design of visible light-responsive photocatalysts in order to effectively utilize solar spectra. However, traditional semiconductor photocatalysts such as TiO_2_ have a large bandgap and low utilization of solar energy, which limits their application in visible-light catalysis. Therefore, in the process of searching for stable and efficient semiconductor photocatalysts with visible activity, a polymer semiconductor graphitic carbon nitride (g-C_3_N_4_), due to its easy synthesis, superior electronic band structure, good stability, and chemical properties, is considered as the next-generation photocatalyst and has aroused widespread interest in the research field.

In this review paper, we will introduce and summarize the basic properties, research history, and recent progress of g-C_3_N_4_, then give some research clues about the structural modification of g-C_3_N_4_ for photocatalytic hydrogen production from water splitting under visible light by using morphological control at the nano scale and electronic band tailoring at the atomic scale.

## 2. Photocatalytic Hydrogen Production

The definition of photocatalysis refers to the change in the rate of chemical or initial reactions of reactants caused by the absorption of photon energy by photocatalysts under the irradiation of ultraviolet, visible, or infrared light, and the resulting chemical changes in reactants. Its essence is a photochemical reaction carried out under the action of a catalyst, which converts solar energy into chemical energy. The main applications of photocatalytic technology include the following: photocatalytic hydrogen production from water [9], carbon dioxide reduction [10], nitrogen fixation [11], reduction of O_2_ to H_2_O_2_ [12], degradation of pollutant staining [13], bacterial disinfection [14], air purification [15], etc. Nowadays, photocatalytic hydrogen production technology is receiving increasing attention from researchers around the world.

**Figure 1:**
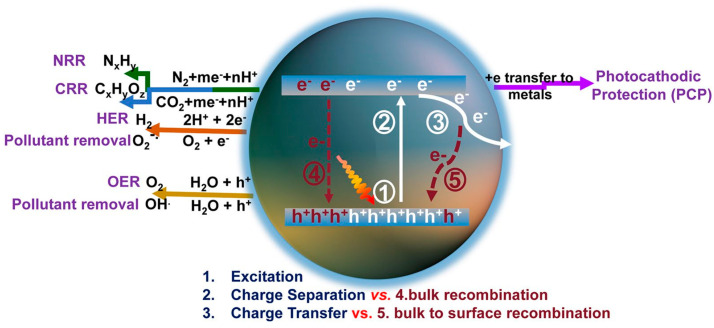
Schematic of photoexcitation, charge transport, and solar applications for g-C_3_N_4_ [16].

Photocatalysts are commonly composed of semiconductor materials. The underlying principle of photocatalytic processes in various research applications is illustrated in Figure 1 [16]. When photons with an energy exceeding the semiconductor’s bandgap width strike the material, electrons in the valence band are excited and promoted to the conduction band (CB), leaving behind holes in the valence band (VB). Subsequently, these photogenerated charge carriers migrate to the catalyst surface, where photogenerated electrons reduce H^+^ in water to produce H_2_. For photocatalytic hydrogen production materials, the potential of their VB and CB is crucial. In order to meet the thermodynamic requirements for photocatalytic water splitting, the CB of the catalyst must be more negative than the oxidation reduction potential of H^+^/H_2_ (0 V vs. NHE), and the VB must be corrected compared to the oxidation reduction potential of O_2_/OH^−^ (1.23 V vs. NHE) [17,18].

**Figure 2:**
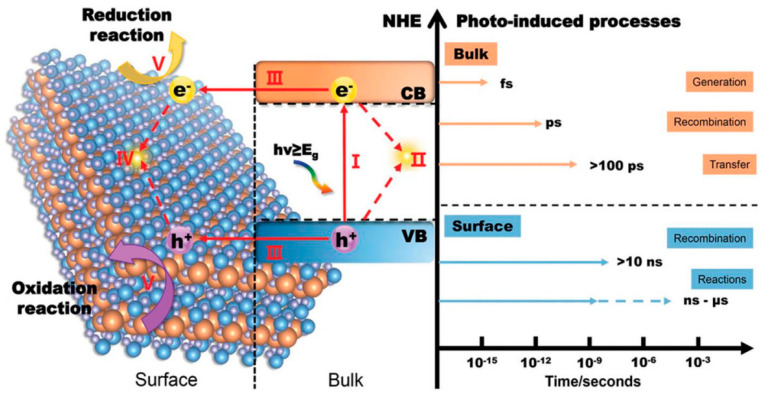
The different time scales of water splitting with semiconductor-based photocatalysis: (I) generation of electron–hole pairs in the bulk of photocatalyst (within several fs); (II) recombination of electrons and holes in the bulk (within a few of ps); (III) separation of excited electron–hole pairs and their transfer to the surface of photocatalyst (hundreds of ps); (IV) recombination of electrons and holes on the surface (tens of ns); (V) participation of the charges in catalytic reactions (several ns to several µs) [20].

In practical scenarios, only a fraction of the photogenerated charge carriers actually engage in surface catalytic reactions. This is because the positively charged holes and negatively charged electrons are drawn to each other and tend to quickly recombine due to the influence of Coulomb force. Here is a more detailed explanation of the kinetic process of photocatalytic reactions on a time scale (Figure 2): Electrons are excited to the conduction band within a few femtoseconds (10^−15^ s) after absorbing light energy. The electrons excited into the conduction band and the holes in the valence band will form a bound state (exciton) due to the interaction of Coulomb force. Excitons move freely within the material through diffusion and begin to de-excite through radiative or non-radiative recombination after a few picoseconds (10^−12^ s). Generally speaking, after exciton dissociation, photogenerated charge carriers can migrate to the catalyst surface after more than 100 ps. The photogenerated electrons and holes that reach the surface of the material will undergo backward excitation after more than 10 ns, or undergo redox reactions with surface adsorbates after several nanoseconds (10^−9^ s) to several microseconds (10^−6^ s). From the above process alone, it can be recognized that in order to improve the catalytic hydrogen production ability of photocatalysts themselves, the following aspects can be approached. (1) Adjust the band structure of semiconductors. On the premise of meeting the thermodynamic requirements of photocatalytic water splitting, a smaller bandgap width can enable the material to absorb more photons of different wavelengths, resulting in a wider range of light response. However, an excessively positive conduction band potential can also reduce the reduction ability of photogenerated electrons, resulting in slower reactions with surface-adsorbed H^+^, thereby intensifying the recombination of surface electrons and holes, ultimately affecting hydrogen evolution activity. (2) Improve exciton dissociation. An excellent exciton dissociation ability can significantly increase the concentration of photogenerated charge carriers and maximize the number of charges. (3) Accelerate the migration of charge carriers. During the migration of charge carriers, electrons and holes are also prone to recombination or capture by defects, resulting in charge loss. (4) Reduce the grain size and increase the specific surface area. On the one hand, more exposed interfaces can increase the number of photons absorbed by the semiconductor and the number of H^+^ ions in contact with the catalyst. A smaller grain size can shorten the migration path of photogenerated carriers. On the other hand, because photogenerated charges are more stable at the interface than in the bulk phase, a larger specific surface area can also extend the lifetime of photogenerated carriers and reduce radiative recombination [19,20].

The research on photocatalytic hydrogen production can be traced back to 1972, when Fujishima A. et al. discovered that the n-type semiconductor TiO_2_, as an electrode for photoelectrochemical cells, can achieve the electrochemical decomposition of water [6]. Subsequently, researchers conducted extensive and in-depth studies on TiO_2_ [21,22,23], but the large bandgap width (3.2 eV) and severe photogenerated carrier recombination rate have always constrained the practical application of TiO_2_. In order to obtain high-performance photocatalysts, various new semiconductor photocatalytic materials have been successively discovered, including the following categories: metal oxides (TiO_2_ [24], ZnO [25], NiO [26], Fe_2_O_3_ [27], Cu_2_O [28], etc.), metal sulfides (CdS [29], ZnS [30], MoS_2_ [31], In_2_S_3_ [32], Zn_x_Cd_1−x_Se [33], etc.), metal phosphides (Ni_2_P [34], GaP [35], etc.), metal nitrides (GaN [36], Ge_3_N_4_ [37], etc.), metal carbides (V_2_C [38], Ti_3_C_2_ [39], etc.), organic framework compounds, metal–organic frameworks [40,41], covalent organic frameworks [42,43], and non-metallic semiconductors (g-C_3_N_4_, elemental carbon [44,45], elemental phosphorus [46,47], etc.). However, these materials all have some problems to varying degrees, such as most catalysts only being active in the ultraviolet region; poor acid and alkali resistance; difficulty in the modification of oxide semiconductors; photo-corrosion and the self-oxidation of metal sulfides, phosphides, etc.; the poor structural stability of metal organic frameworks; and so on. Therefore, the development of photocatalysts with a good visible-light response, high hydrogen evolution activity, good stability, low preparation cost, and no environmental pollution has always been a research focus.

## 3. Graphitic Carbon Nitride Photocatalyst

### 3.1. The Development of Graphitic Carbon Nitride

**Figure 3:**
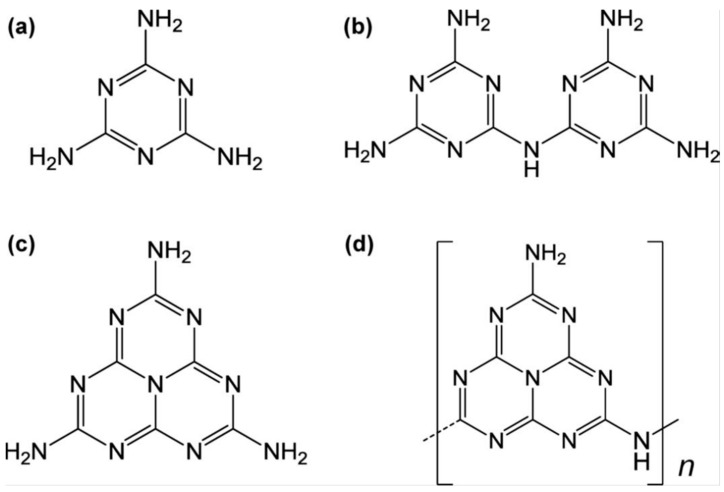
Chemical structure of (**a**) melamine, (**b**) melam, (**c**) melem, and (**d**) melon [52].

Carbon nitride, a venerable non-metallic semiconductor, has its origins in the early 19th century. As early as 1834, Liebig and Berzelius [48] synthesized a substance known as melon through the pyrolysis of ammonium chloride and potassium thiocyanate. They also documented the existence of melamine, melam, melem, and melon (as shown in Figure 3), compounds that were later recognized as being based on triazine or tri-s-triazine molecular structures. However, at the time of their discovery, the precise molecular formulas of these compounds remained undetermined. In 1922, Franklin [49] studied the relevant compounds and believed that the final polymerization product of heated melon was (C_3_N_4_)_x_, and he estimated the possible chemical structure. In 1937, Pauling and Sturdivant [50] proposed through X-ray crystallography that triazine is the basic structure of melon. Subsequently, Redemann and Lucas [51] further speculated on Franklin’s C_3_N_4_ and found that the structure of melon was not singular but a mixture of various carbon nitrogen polymers. These macromolecules, formed through condensation reactions, were planar structures composed of nitrogen atoms linked by heptazine rings.

**Figure 4:**
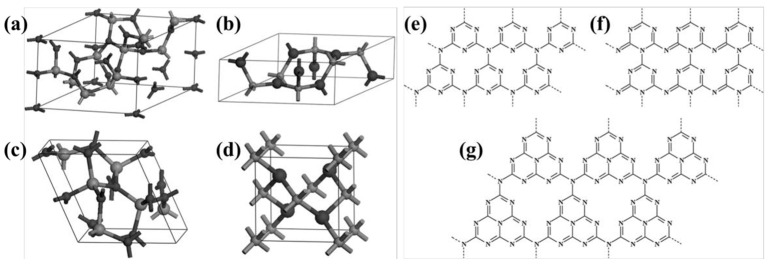
Primitive cells of (**a**) α-C_3_N_4_, (**b**) β-C_3_N_4_, (**c**) cubic C_3_N_4_ and (**d**) pseudocubic C_3_N_4_ (carbon and nitrogen atoms are depicted in big and small balls, respectively) [58], (**e**) g-h-triazine (s-triazine-based hexagonal structure), (**f**) g-o-triazine (s-triazine-based orthorhombic structure), and (**g**) g-h-heptazine (tri-striazine/heptazine-based hexagonal structure) [59].

However, C_3_N_4_, derived from melon, had long been neglected due to its chemical inertness and the uncertainty surrounding its composition. It was not until 1982 that Leonard et al. [53] first described the crystal structure of cyanuric acid derivatives, which were found to be arranged in a co-planar configuration, thus validating Pauling’s earlier conjecture. With the development of first-principles density functional theory (DFT) calculations, Liu and Cohen [54] simulated the substitution of Si atoms with C atoms in β-Si_3_N_4_ in 1989. The calculations showed that β-C_3_N_4_ had a bulk modulus comparable to or greater than diamond and suggested the possibility of synthesizing this material in the laboratory, which sparked research interest in C_3_N_4_. In 1996, Teter and Hemley [55] proposed five possible structures for C_3_N_4_ through calculations (Figure 4)—β-C_3_N_4_, α-C_3_N_4_, graphitic-phase C_3_N_4_ (g-C_3_N_4_), quasi-cubic-phase C_3_N_4_, and cubic-phase C_3_N_4_—and pointed out that α-C_3_N_4_ and g-C_3_N_4_ are more stable in energy. Among them, g-C_3_N_4_ has been widely studied due to its ease of synthesis. At that time, researchers had already proposed structures for g-C_3_N_4_ based on triazine and heptazine units, both of which are part of the hexagonal crystal system. However, it was the triazine-based structures that were more extensively reported and characterized in detail [56]. In 1999, Alves et al. [57] reported a new g-C_3_N_4_ structural model, which is based on a new order of carbon vacancies and has an orthorhombic crystal cell. Since then, there have been three types of g-C_3_N_4_, and C_3_N_4_ mainly has seven theoretical structures (Figure 4). Nevertheless, varying chemical structures are associated with differing levels of stability. Kroke et al. [56] determined through computational analysis that the triazine structure is the most probable to exist, and it is approximately 30 kJ/mol more stable than the previously reported lowest energy phase of C_3_N_4_. Xu et al. [58] calculated all phases of C_3_N_4_ and found that g-C_3_N_4_ based on the heptazine ring (triazine structure) has an energy advantage compared to other phases. Only the quasi-cubic phase of C_3_N_4_ and g-C_3_N_4_ based on a triazine structure with an hexagonal crystal system have direct band gaps, while the rest of the structures are indirect band gap semiconductors. Most of the current work has shown that nitrogen-rich precursors such as condensed cyanamide, melamine, urea, or thiourea are synthesized based on the triazine structure of g-C_3_N_4_, and the academic community generally believes that the basic structural unit of g-C_3_N_4_ is triazine. Therefore, the g-C_3_N_4_ described later is based on the triazine structure, namely the heptazine ring [52].

The utilization of g-C_3_N_4_ dates back to 2008, when Thomas et al. [60] discovered that this organic semiconductor polymer is capable of catalyzing Friedel–Crafts reactions, the trimerization reactions of triple bonds, and even the decomposition of CO_2_.These reactions could only be carried out using precious metals or transition metals in the past, which opened up the application of g-C_3_N_4_ in the field of catalysis. In 2009, Wang et al. [61] made the groundbreaking discovery that g-C_3_N_4_ could generate hydrogen (H_2_) from water under visible-light irradiation with the aid of a co-catalyst, thereby paving the way for research into the photocatalytic hydrogen production capabilities of g-C_3_N_4_.

### 3.2. Structure and Properties of Graphitic Carbon Nitride

**Figure 5:**
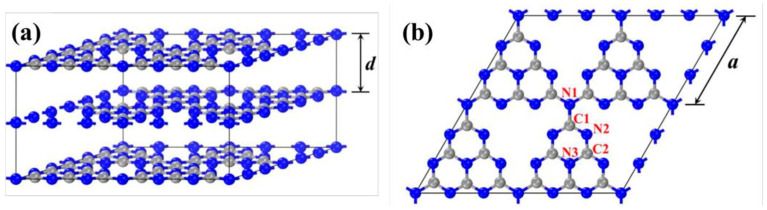
Geometric structure of g-C3N4 (the gray and blue balls are carbon and nitrogen atoms, respectively) (**a**) bulk g-C_3_N_4_ and (**b**) single layer g-C_3_N_4_ [59].

g-C_3_N_4_ is named after its graphite-like layered stacking structure, with a theoretical interlayer spacing d of 3.19 Å (Figure 5a). A density functional theory computation performed by the CASTEP module in Materials Studio is an effective way to investigate the band structures and electronic and optical properties of g-C_3_N_4_. Each layer is composed of triazine units connected by nitrogen atoms extending outward, and the side length of the triangular cavity formed by three heptazine rings is 7.14 Å (Figure 5b) [62,63]. There are three different chemical environments of nitrogen atoms and two different chemical environments of carbon atoms in the structure, denoted as N1, N2, N3, C1, and C2. Among them, C1, C2, and N2 are sp^2^-hybridized, and N1 and N3 are sp^3^-hybridized. The bond lengths of N1–C1, C1–N2, N2–C2, and C2–N3 are 1.47 Å, 1.34 Å, 1.33 Å, and 1.39 Å, respectively [64]. The p_z_ orbitals of the sp^2^-hybridized carbon and nitrogen atoms in the heptazine ring overlap with each other, forming delocalized π bonds similar to benzene rings. The stacking mode of the original g-C_3_N_4_ layers is of the “AB” type, and the interaction energy between adjacent layers is 0.036 eV·Å^−2^, corresponding to the van der Waals forces caused by weak π-π interactions between layers [65,66].

**Figure 6:**
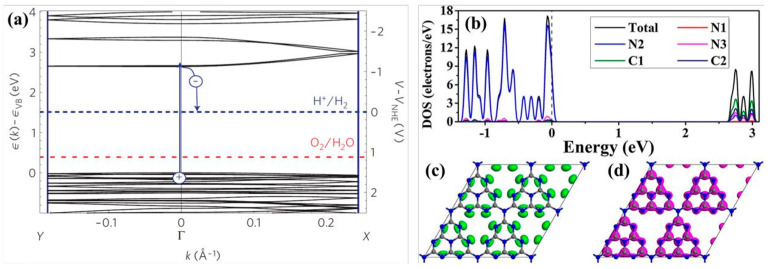
(**a**) Electronic structure of g-C_3_N_4_ [61], (**b**) density of states (DOS) of g-C_3_N_4_, (**c**) highest occupied molecular orbital (HOMO), and (**d**) lowest unoccupied molecular orbital (LUMO) of monolayer g-C_3_N_4_ [59].

The generalized gradient approximation with the Perdew–Burke–Ernzerhof (GGA-PBE) functional is commonly employed to evaluate the bandgap of g-C_3_N_4_. After extensive theoretical calculations and experimental verification, the bandgap width of pristine g-C_3_N_4_ is determined to be approximately 2.7 eV (Figure 6a), which corresponds to a light absorption edge of around 460 nm. This bandgap places the reduction potential of H^+^/H_2_ and the oxidation potential of O_2_/OH^−^ well within the bandgap of g-C_3_N_4_, thereby fully satisfying the thermodynamic conditions necessary for the absorption of visible light and photocatalytic water splitting to produce hydrogen and oxygen [67]. The VB edge of g-C_3_N_4_ is predominantly constituted by N2 atoms, whereas the CB edge is primarily composed of C1 and C2 atoms, with subsequent contributions from N2 and N3 atoms, as depicted in Figure 6b. When delving into the atomic orbitals, the VB edge is populated by the 2p orbitals of nitrogen, and the CB edge is populated by the 2p orbitals of both carbon and nitrogen. This finding aligns with the pattern where elements with a higher electronegativity tend to form the VB edge [64]. Thus, in photocatalytic hydrogen production reaction, N2 atoms serve as the sites for both oxidation and reduction reactions, while C1, C2, and N3 atoms act as reduction sites. However, N1 atoms scarcely contribute to the formation of the VB and CB edges, meaning that electrons are neither generated nor excited to N1 atoms. This lack of involvement hinders the transfer of photoelectrons between heptazine rings, leading to a high recombination rate of electron–hole pairs in the pristine g-C_3_N_4_ [66,68]. Figure 6c,d show the highest occupied molecular orbital (HOMO) and lowest unoccupied molecular orbital (LUMO) locations of monolayer g-C_3_N_4_. The occupied and unoccupied orbits are almost uniformly delocalized over the heptazine units, due to the high symmetry and repeatability of the in-plane molecular structure without any distortion. This result could well explain the high recombination rate between photogenerated electrons and holes in pristine g-C_3_N_4_.

Thermogravimetric analysis (TGA) of g-C_3_N_4_ indicates that even at 600 °C in an air atmosphere, the pristine g-C_3_N_4_ demonstrates excellent stability and non-volatility. At 630 °C, a pronounced endothermic peak emerges, accompanied by a continuous loss in weight, due to the thermal decomposition of g-C_3_N_4_ into nitrogen and cyanide-containing fragments. Beyond 700 °C, g-C_3_N_4_ completely vanishes, fully vaporizing into CO_x_ and NO_x_ gases [52]. While varying preparation methods can influence the thermal stability of g-C_3_N_4_, it still ranks among the most thermally stable organic materials, surpassing all high-temperature polymers in this regard. Due to van der Waals forces between layers, g-C_3_N_4_ has not been detected to be soluble or reactive in most traditional solvents, including water, alcohol, DMF, THF, ether, toluene, etc. The exceptional chemical and thermal stability of g-C_3_N_4_ allows photoelectrochemical cells to function effectively in an oxygen-rich environment, a capability that is quite rare among such materials [19].

### 3.3. Synthesis Pathways of Graphitic Carbon Nitride

**Figure 7:**
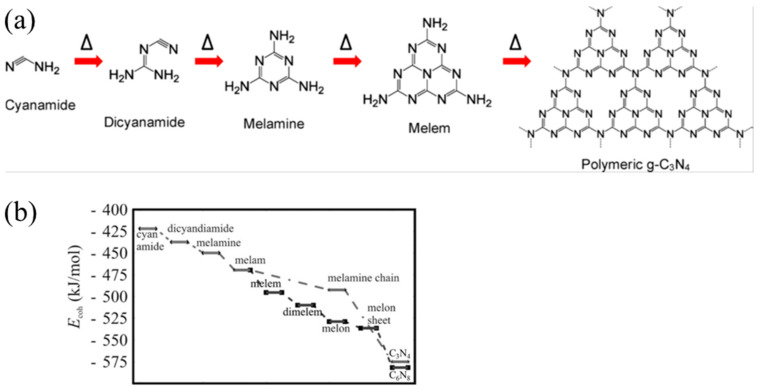
(**a**) Reaction pathway for the development of g-C3N4 using cyanamide as the precursor (the delta symbols and arrows illustrate the reaction temperature/time increasing) [52], (**b**) calculated energy diagram for the synthesis of carbon nitride [60].

Typically, g-C_3_N_4_ could be prepared via various inexpensive nitrogen-rich precursors such as cyanamide, melamine, urea, and thiourea [69]. Other materials like formamide [70], guanidine hydrochloride [71], guanidine carbonate [72], and 3-amino-1,2,4-triazole [73,74] have also been reported. The most widely used methods are thermal condensation [75], solvothermal [76], plasma sputtering reaction deposition [77], chemical vapor deposition (CVD) [78], etc. DFT calculations also can be used to explore the synthesis mechanism of g-C_3_N_4_ from precursors [79]. Taking cyanamide as an example, the conventional synthesis pathway for g-C_3_N_4_ could be summarized in the following steps (Figure 7a): Initially, cyanamide molecules undergo condensation to form dicyandiamide and melamine at temperatures of 203 °C and 234 °C, respectively. At approximately 335 °C, all these precursors have been converted into melamine-based products. Subsequently, melamine undergoes rearrangement accompanied by the release of ammonia. The properties of the final product in this synthesis can vary based on whether the reaction is carried out in a sealed or unsealed environment. At 390 °C, melem units are formed (Figure 3c). Subsequently, these melem units initiate condensation to form melon networks, which may eventually lead to the formation of polymeric g-C_3_N_4_ at 520 °C. Alternative precursors, including urea and thiourea, similarly undergo an initial transformation into melamine. The subsequent synthesis steps for these materials follow a pattern that is consistent with the process previously described. A significant challenge in this synthesis process is that melamine gradually sublimates at elevated temperatures. However, the sublimation of melamine can be significantly mitigated when it is in the presence of other substances [80], particularly hydrogen-bonding (H-bridges) donors. Directly heating melem above 500 °C further releases ammonia gas, resulting in g-C_3_N_4_ with a higher degree of polymerization.

In order to gain a more precise understanding of the reaction pathway, Thomas et al. [60] employed simulation calculations to delve into the steps of this reaction. The findings reveal (Figure 7b) that melamine initially condenses into the metastable intermediate melam (Figure 3b) in a pairwise manner. Subsequently, melam can undergo condensation through two distinct pathways: One pathway is the triazine route (indicated by the dotted line), which initially leads to the formation of melamine chains and ultimately results in the generation of C_3_N_4_. The other type is the triazine pathway (dashed line), which initially forms melem and then condenses into melon (as shown in Figure 3d), eventually yielding C_6_N_8_. Notably, the triazine pathway possesses a higher binding energy, which is also the reason why heptazine-based g-C_3_N_4_ is commonly produced in experiments. The final stage involves the fusion of melom chains to form g-C_3_N_4_ flakes.

## 4. Modification of Graphitic Carbon Nitride

Despite its numerous inherent advantages, g-C_3_N_4_ faces several challenges: its limited visible-light absorption region, low electrical conductivity, high recombination rate of photogenerated electrons and holes, and the tendency of thermally polymerized g-C_3_N_4_ to aggregate, leading to a low specific surface area and a scarcity of sufficient active sites. These shortcomings still constrain the practical application of g-C_3_N_4_. Fortunately, varying synthesis conditions—such as the choice of reactants and the conditions under which the reaction takes place—substantially influence the physicochemical properties and performance of the resulting g-C_3_N_4_. Enhanced catalytic activity can typically be attributed to and elucidated experimentally through structural, optical, and photoelectrochemical perspectives [81]. This section will elaborate on the modification methods of g-C_3_N_4_ from two perspectives: external morphology and internal structure.

### 4.1. Nano-Morphological Control

**Figure 8:**
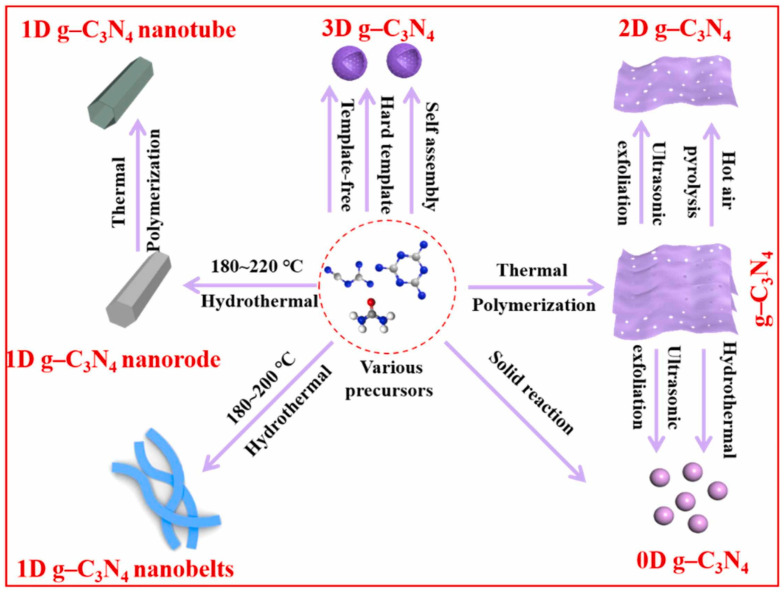
Schematic illustration of the techniques for synthesis of different-dimensional g-C_3_N_4_ photocatalysts (0–3D) [82].

Controlling the shape of nanomaterials is pivotal for enhancing their specific surface area, which in turn significantly influences their physicochemical properties. g-C_3_N_4_ can be classified into structures ranging from zero-dimensional to three-dimensional based on its size (Figure 8) [82]. The key to achieving this lies in selecting the appropriate methods and parameters. The size of zero-dimensional g-C_3_N_4_ or g-C_3_N_4_ quantum dots is usually less than 20 nm, which can be synthesized using hydrothermal methods [83,84], the ultrasonic exfoliation method [85,86], and solid-state reaction method [87]. Zero-dimensional g-C_3_N_4_ has great application prospects in biological imaging, optical sensors, and other fields due to its excellent fluorescence quantum efficiency, but there are few reports on hydrogen production.

Typical one-dimensional g-C_3_N_4_ includes g-C_3_N_4_ nanorods [76,88,89] and nanotubes [90,91,92]. Unlike conventional synthesis methods, Li et al. [93] prepared g-C_3_N_4_ nanorods using the infrared heating of melamine without the need for templates or additional organic compounds. They found that the precursor would assemble into nanorods at low power levels and grow into nanoplates at high power levels. Among the various forms, g-C_3_N_4_ nanorods have demonstrated a superior hydrogen production performance compared to nanoplates. This is attributed to their highly concentrated and oriented growth direction, which enhances the separation of photogenerated charge carriers. Additionally, the presence of structural defects is significantly diminished in these nanorods. Compared to nanorods, the hollow tubular structure of one-dimensional nanotubes offers several advantages. It enhances the material’s light absorption and scattering capabilities, and it significantly boosts the specific surface area, which is beneficial for photocatalytic reactions. Shalom et al. [94] directly calcined needle-shaped self-assembled precursors of melamine and cyanuric acid in chloroform solvent under a nitrogen atmosphere to obtain a tubular g-C_3_N_4_. Guo et al. [92] obtained regular hexagonal rod-shaped precursors by the hydrothermal treatment of a mixed solution of phosphoric acid and melamine. The hexagonal prisms of g-C_3_N_4_ were vertically stacked from nanosheets, and the self-assembled precursors were then calcined to yield hexagonal tubular g-C_3_N_4_. In contrast to the supramolecular self-assembly strategy, the template method, while more cumbersome, can prepare ultra-long and ordered g-C_3_N_4_ nanotubes. This method allows for precise control over the shape and size of the nanorods, which is beneficial for various applications. The template method involves using a structural framework that guides the formation of g-C_3_N_4_ nanorods with the desired properties, which is a significant advantage over self-assembly in terms of achieving a specific morphology. Wu et al. [95] employed a CVD method to prepare one-dimensional g-C_3_N_4_ nanotubes on SiO_2_ nanofibers. The nanotubes had a diameter of approximately 300 nm and a length of 6 μm. The surface of the prepared nanotubes was smooth, and they possessed a significantly higher aspect ratio compared to similar reports. Moreover, the hydrogen production performance reached 4605.2 μmol h⁻^1^ g⁻^1^.

Two dimensional g-C_3_N_4_, also known as g-C_3_N_4_ nanosheets or nanofilms, is typically exfoliated into nanosheets using methods such as thermal exfoliation [96], chemical exfoliation [97], and ultrasound-assisted liquid-phase exfoliation [98]. This is also a commonly used and effective way to increase the specific surface area. A typical instance is that Dong et al. [99] carried out a second calcination on the synthesized block-shaped g-C_3_N_4_ by using the thermal exfoliation method and ultimately obtained porous g-C_3_N_4_ nanosheets. They discovered that as the exfoliation temperature increased, the color of the sample gradually became lighter, and the layer thickness and size of the nanosheets progressively decreased. Moreover, due to the influence of the quantum size effect, the band structure of the nanosheets could also be continuously adjusted. In addition to the top-down approach mentioned above, g-C_3_N_4_ nanosheets can also be obtained through a bottom-up approach (from precursor to product). Chen et al. [78] grew ultra-thin ordered carbon nitride films on metal substrates using the CVD method, with a film thickness of about 10 nm. Tong et al. [100] used chemical exfoliation to obtain g-C_3_N_4_ nanosheets with a thickness of approximately 2.5 nm and an average diameter of 80 nm by adding water to a mixed solution of bulk g-C_3_N_4_ and concentrated sulfuric acid. Zhang et al. [101] obtained white ultra-thin g-C_3_N_4_ nanosheets by ultrasonicating yellow block-shaped g-C_3_N_4_ in water. The thickness of the prepared g-C_3_N_4_ nanosheets was about 2.5 nm, and the Tyndall effect was observed in their aqueous solution. Three-dimensional g-C_3_N_4_ has shown great potential in engineering applications due to its excellent mass transfer ability and ease of recycling. Common synthesis methods include the hard template method [102,103], soft template method [104,105], molecular self-assembly method [106], and hydrothermal method. Wu et al. [107] utilized monodispersed SiO_2_ nanospheres prepared through the hydrolysis method as templates and ammonium hydrogen fluoride as the template remover to fabricate three-dimensional honeycomb g-C_3_N_4_ by the CVD method. Although g-C_3_N_4_ prepared with hard templates has a more stable and ordered three-dimensional structure, soft templates have also drawn attention because they are easier to remove and the preparation process is relatively simple. Zhao et al. [108] took advantage of the supramolecular self-assembly of melamine and cyanuric acid and the structure-oriented properties of ionic liquids to synthesize high-specific-surface-area and high-porosity hollow mesoporous g-C_3_N_4_ spheres by a one-step soft template method. They discovered that the prepared hollow g-C_3_N_4_ spheres had a large number of mesopores, which were formed due to the release of volatile structural domains and the decomposition of ionic liquids. Wang et al. [109] prepared g-C_3_N_4_ microspheres by a hydrothermal method with dicyandiamide and melamine as precursors. They were able to control morphology, band structure, and defect content by simply adjusting the concentration of the precursors.

### 4.2. Electronic Band Tailoring

Although nano-morphology control has significantly enhanced the hydrogen production performance of g-C_3_N_4_, its effect mainly lies in increasing the specific surface area and the changes in quantum, optical, and other physical properties brought about by the special morphology. However, the design of typical nanostructures makes it difficult to effectively regulate the intrinsic structure of g-C_3_N_4_ at the molecular level. Therefore, it is essential to regulate the internal structure of g-C_3_N_4_ via a suitable strategy. Defect engineering, which involves the intentional introduction of impurities into the matrix or the modulation of atomic periodicity in semiconductors, has been proven to be an efficient strategy for tailoring the electronic band structures, optical properties, and conductivity of g-C_3_N_4_. Below, we will elaborate on three types of structural control methods: carbon or nitrogen vacancy, element doping, and chemical functional group modification.

#### 4.2.1. Carbon/Nitrogen Vacancy

The vacancy defects in the g-C_3_N_4_ structure only include the absence of carbon [110,111] and nitrogen [112] atoms in its heptazine framework. Gao et al. [113] directly calcined urea aqueous solution and obtained ultra-thin g-C_3_N_4_ nanosheets containing carbon vacancy through in situ exfoliation by endogenous gas. Through simulation calculations and experimental tests, they found that carbon defects make the CB and VB positions of g-C_3_N_4_ more negative, thereby enhancing the reduction ability of photogenerated electrons. Compared with carbon vacancy, the impact of nitrogen vacancy on g-C_3_N_4_ is more complex. This is because nitrogen atoms at different positions make significant contributions to the band structure of g-C_3_N_4_. Moreover, due to differences in electronegativity, the delocalized electrons in the heptazine ring are more inclined towards sp^2^-hybridized nitrogen atoms. In the former section, it is discussed that nitrogen defects typically contribute to reducing the bandgap and act as active sites for catalytic reactions. Additionally, due to the creation of localized states, nitrogen defects often result in an upward shift in the light absorption tail. This can lead to significant energy level splitting and the introduction of an intermediate energy level within the bandgap [114,115,116]. However, an excessive number of nitrogen defects, particularly the lack of bridging nitrogen atoms between heptazine rings, can adversely affect the conductivity of g-C_3_N_4_ [117]. Wang et al. [118] synthesized nitrogen-rich porous g-C_3_N_4_ by calcining a supramolecular complex of KOH, melamine, and cyanuric acid. They discovered that the prepared sample exhibited a more negative Zeta potential, indicating a higher concentration of negative charges on its surface, which could interact with more H^+^ ions.

#### 4.2.2. Element Doping

**Figure 9:**
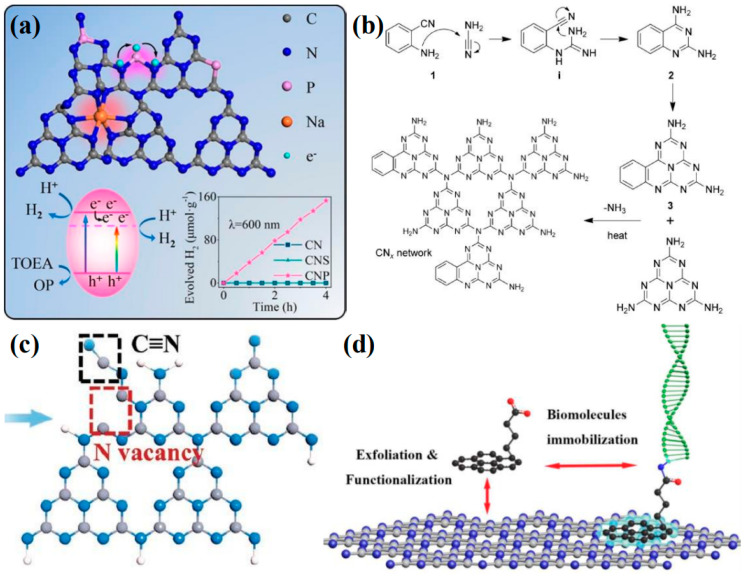
(**a**) Proposed doping sites of P and Na and their effects on the lattice structure of g-C_3_N_4_ (the H_2_ evolution mechanism and properties of the prepared sample are shown below) [128], (**b**) the copolymerization of dicyandiamide/cyanamide with 2-aminobenzonitrile [129], (**c**) the defect structure model of Nv−C≡N−CN [130], (**d**) schematic diagram of Py−COOH-modified g-C_3_N_4_ trapping molecular probe [131].

Element doping is recognized as a straightforward and potent method for modifying the intrinsic electronic properties of materials. The doping type can be categorized into gap doping and substitution doping, depending on the specific doping sites. Substitution doping is a common occurrence when the size of the doped atoms is similar to that of the carbon and nitrogen atoms in the heptazine unit. Through orbital hybridization, these doped atoms form covalent bonds with the local atoms, which significantly and intrinsically alter the chemical state and band structure of g-C_3_N_4_. Typically, if the electronegativity of the dopant is lower than that of carbon and nitrogen (such as sulfur [119], phosphorus [120], or boron [121]), it will induce a negative shift in the CB minimum. Conversely, if the electronegativity of the dopant is higher than that of carbon and nitrogen (such as fluorine [122], chlorine [123], or oxygen [124]), it will result in a positive shift in the VB maximum [117]. Even when dopants induce significant disorder in the lattice potential energy of g-C_3_N_4_, the CB and VB will still extend into the bandgap, creating an intermediate bandgap. When the dopants have a larger atomic radius, such as most metal atoms, they are typically anchored within the interlayer spaces or triangular cavities of g-C_3_N_4_, resulting in a form of gap doping. Owing to the enhanced delocalization of valence electrons in these doped atoms, they frequently alter the charge density within g-C_3_N_4_, thereby influencing the mobility of charge carriers. However, disparities in atomic size and electronegativity can lead to substantial lattice distortions. In addition to single-element doping, the beneficial effects of multiple dopants can be harnessed through the approach of co-doping [125,126,127]. For example, Wang et al. [128] reported a method for co-doping P and Na into g-C_3_N_4_ using a post-heat treatment process (Figure 9a). The P atoms not only substitute for the sp_2_-hybridized carbon and nitrogen atoms within the heptazine rings but also facilitate the formation of N-P-N bridging bonds between adjacent heptazine rings, thereby enhancing the transfer of charge carriers. Consequently, the incorporation of P atoms introduces an intermediate bandgap into the material, which substantially broadens the range of visible-light absorption. Meanwhile, Na^+^ is doped into the triangular cavities, contributing additional electrons to the π-conjugated system and inducing significant lattice distortion.

#### 4.2.3. Chemical Functional Group Modification

The molecular structure of polymeric g-C_3_N_4_ allows for the adjustment of its electronic properties through minor modifications to the molecular structure during the copolymerization process. This can be achieved in conjunction with structure-matching organic additives. Commonly modified functional groups for g-C_3_N_4_ include the -C≡N [132,133], -OH [134], and -CH_3_ [135], among others. These functional groups typically enhance the separation of photogenerated charge carriers, promote electron excitation and conduction, and may even reduce the energy barriers associated with doping or reactions. Zhang et al. [130] successfully incorporated the -C≡N and nitrogen vacancies into g-C_3_N_4_ using a simple two-step process (Figure 9c). The -C≡N, known for its strong electron-withdrawing properties, facilitates exciton dissociation. Computational studies have indicated that the presence of the -C≡N also enhanced H^+^ adsorption. Naturally, chemical linkages between g-C_3_N_4_ and various molecules fall within this category of modifications. For example, Chu et al. [136] incorporated anthraquinone into the edges of g-C_3_N_4_ flakes and coordinated single cobalt atoms on g-C_3_N_4_. By spatially separating the oxidation and reduction co-catalysts, they significantly enhanced the separation of surface charges. In addition to modifying the molecular structure of g-C_3_N_4_ through single- and double-bond connections, the conjugated system of the heptazine ring can be extended or combined with other carbon–nitrogen heterocycles. This approach is also a common method for functional modification, typically achieved through copolymerization [137]. Zhang et al. [129] synthesized phenyl-modified g-C_3_N_4_ by copolymerizing 2-aminobenzonitrile with dicyandiamide (Figure 9b). The findings indicated that the incorporation of additional aromatic groups led to an expansion of the π-conjugated system in g-C_3_N_4_. Characterization and theoretical calculations revealed that the bandwidth of the resulting product was narrowed, and its optical and electrical properties were enhanced. In addition to the functional groups grafted by chemical bonds within the in-plane heptazine framework in g-C_3_N_4_, the advantage of linking other molecules via intermolecular forces lies in its ability to substantially retain the intrinsic properties of each constituent while synergistically harnessing their unique characteristics. For instance, Ji et al. [131] have demonstrated the modification of the aromatic molecule Py-COOH onto pristine g-C_3_N_4_ through π-π stacking interactions by employing a facile mechanical grinding technique (Figure 9d). This approach not only enhanced the immobilization of g-C_3_N_4_ molecular probes but also preserved the inherent optoelectronic properties of g-C_3_N_4_.

## 5. Modification of Graphitic Carbon Nitride for Photocatalytic H_2_ Production

Photocatalytic H_2_ production serves as a promising and eco-friendly approach to mimic the natural process of plant photosynthesis, offering solutions to both energy scarcity and environmental challenges. g-C_3_N_4_, a metal-free, non-toxic, and highly stable material with a favorable band structure, has emerged as a leading candidate for photocatalytic H_2_ evolution. This process is achieved with the aid of sacrificial reagents and co-catalysts in water. The above descriptions show that the morphology and electronic band of g-C_3_N_4_ can be well regulated to enhance its overall photocatalytic H_2_ production performance. Over the past ten years, there has been a significant advancement in the development of g-C_3_N_4_-based materials, which have demonstrated enhanced photocatalytic performance. In this section, we will take structural control as the starting point to improve the separation efficiency and density of photogenerated carriers of g-C_3_N_4_ through electronic and chemical structure modification. Defects have been strategically incorporated into the g-C_3_N_4_ framework via methods such as element doping, atmosphere calcination, and copolymerization. The causes of defect formation and their underlying mechanisms affecting the photocatalytic performance of g-C_3_N_4_ were carefully discussed. We will focus on a series of works on the photocatalytic H_2_ evolution using g-C_3_N_4_-based photocatalysts.

**Figure 10:**
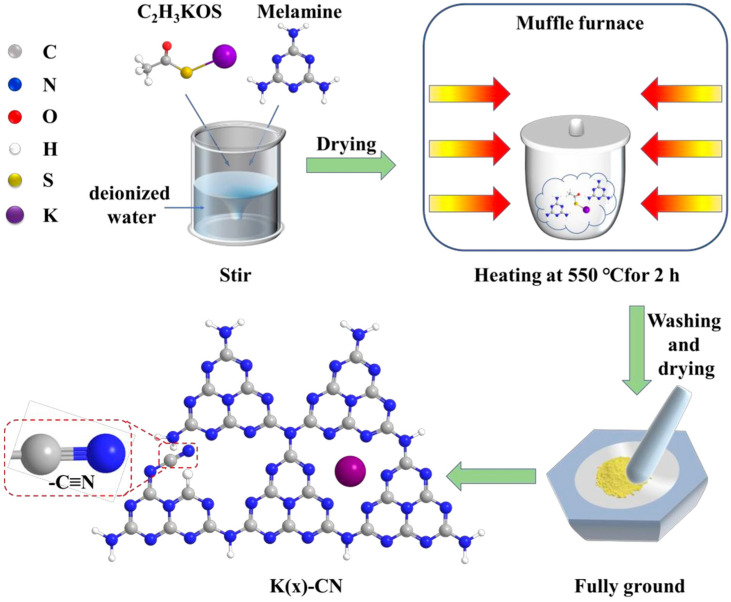
Schematic representation of K(x)-CN preparation process [138].

**Figure 11:**
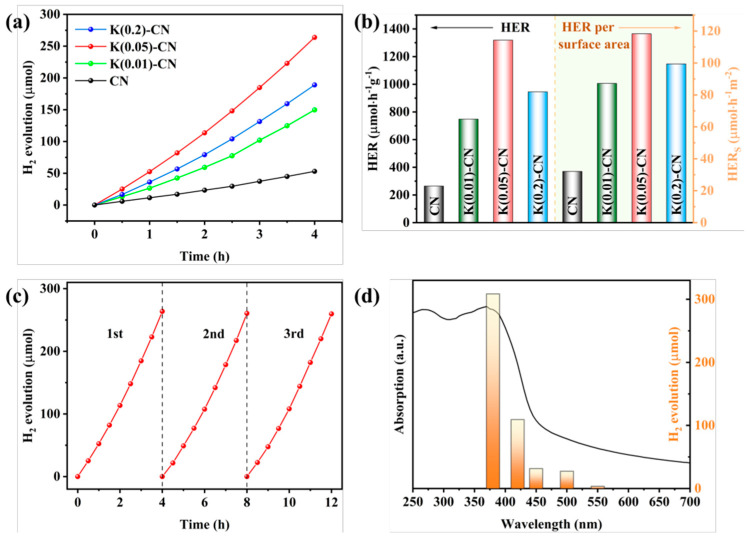
(**a**) Temporal evolution of H_2_ evolution curves and (**b**) HER and HER per surface area of CN and K(x)-CN, (**c**) stability test and (**d**) wavelength-dependent test of the H_2_ evolution of K(0.05)-CN [138].

For instance, through a straightforward and cost-effective doping process, a modified g-C_3_N_4_ material incorporating both K^+^ and -C≡N groups was synthesized, as depicted in Figure 10. The introduction of these groups and their surrounding chemical environment have been meticulously analyzed. The separate impacts of K^+^ and cyanide groups on the performance of g-C_3_N_4_ have been discussed in detail. A comprehensive characterization was performed to assess the changes in the chemical structure, chemical state, bandgap structure, and carrier dynamics of the photocatalyst under the combined influence of these two modifiers. A hypothesis was proposed regarding the intrinsic mechanisms that contribute to the enhancement of catalytic activity and the efficiency of visible-light utilization. Figure 11 illustrates the increased H_2_ production in relation to the surface area of g-C_3_N_4_ modified with both K^+^ and cyanide groups, as well as the material’s good stability and the wavelength-dependent performance of H_2_ production [138].

**Figure 12:**
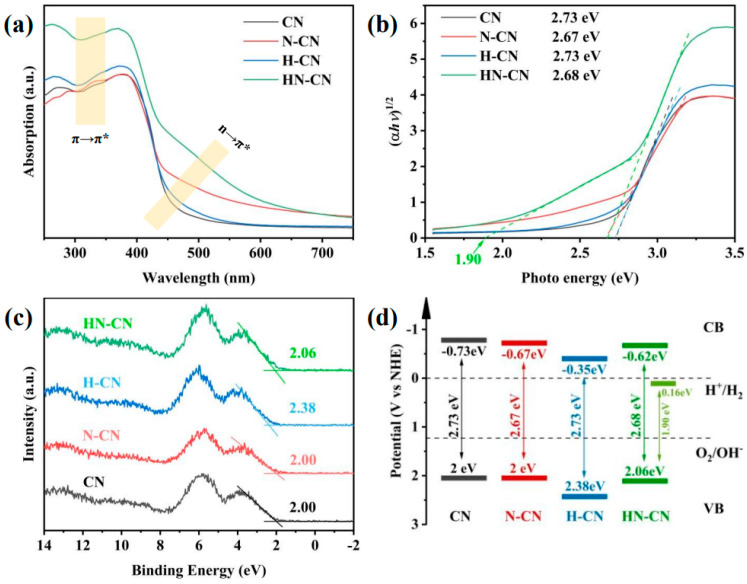
(**a**) UV−Vis DRS spectra, (**b**) plots of converted Kubelka–Munk function vs. photon energy, (**c**) XPS VB spectra, and (**d**) corresponding electronic band structures of CN, N-CN, H-CN, and HN-CN [139].

**Figure 13:**
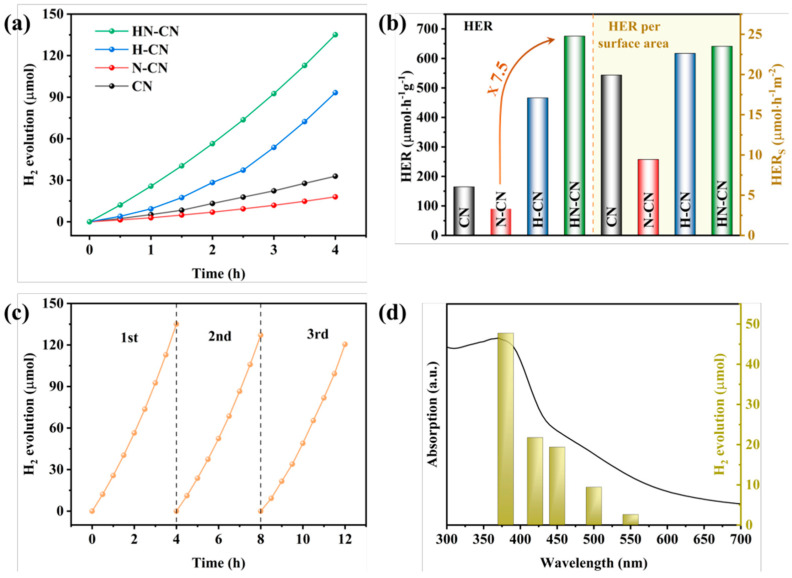
(**a**) Temporal evolution of H2 evolution curves and (**b**) HER and HER per surface area of CN, N-CN, H-CN, and HNCN. (**c**) Stability test and (**d**) wavelength-dependent test of H2 evolution of HN-CN [139].

In order to investigate the root cause of defects in the synthesis of g-C_3_N_4_ under N_2_ atmosphere and to design a method for preparing g-C_3_N_4_ with an excellent H_2_ production performance, we prepared g-C_3_N_4_ with a small amount of high-quality defects and significantly improved H_2_ production performance through a simple protonation pretreatment and secondary calcination under an inert atmosphere. On the one hand, we conducted a thorough investigation into the types of defects generated using advanced characterization techniques such as Fourier-transform infrared spectroscopy (FTIR) and X-ray photoelectron spectroscopy (XPS). This analysis allowed us to propose a chemical synthesis pathway for the formation of defects in g-C_3_N_4_ under a N_2_ atmosphere. On the other hand, we confirmed the efficacy of a protonation treatment on precursors to optimize the hydrogen production performance of g-C_3_N_4_. This treatment not only enhances the polymerization degree of the resulting product but also effectively suppresses the formation of detrimental defects, thereby significantly improving the H_2_ production capabilities of g-C_3_N_4_. Figure 12 and Figure 13 give these tailored electronic band structures and their corresponding H_2_ evolution performances [139].

**Figure 14:**
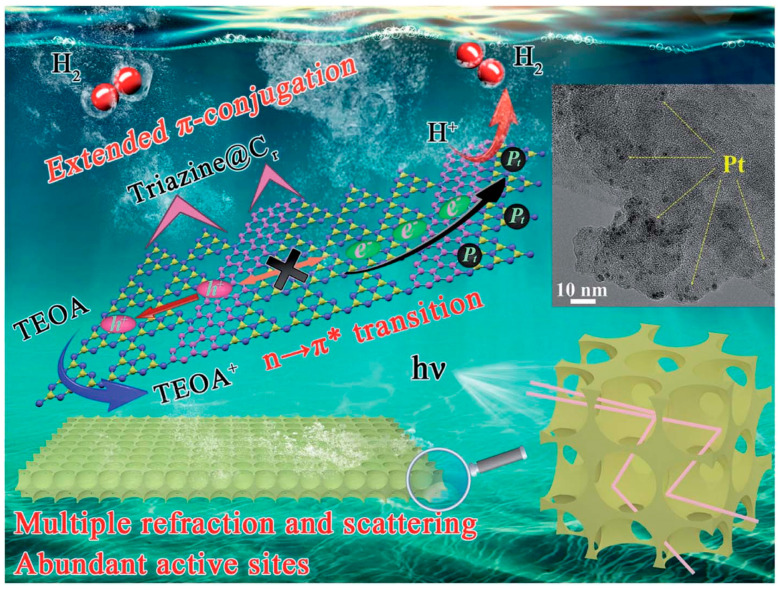
Schematic illustration of the mechanism of the visible-light-induced hydrogen evolution process from water splitting using Cr–PHCN catalysis from nano-scale morphological control and atomic scale electronic band tailoring [107].

Importantly, the control of electronic structure and the design of nano-morphology can be carried out simultaneously in g-C_3_N_4_. For example, the successful fabrication of a multiple-ordered porous honeycomb structural g-C_3_N_4_ through the one-step co-pyrolysis of melamine and glucose in air via the CVD method has led to significant improvements in band edge optimization and carrier transport dynamics [107]. This unique morphology effectively enhances light absorption through multiple internal reflections and scattering, which in turn accelerates the rate of electron transmission. Additionally, the large specific surface area of this structure provides a plethora of active sites, which are crucial for enhancing the efficiency of photocatalytic reactions. Concurrently, the electronic structure is modulated by the in-plane splicing of carbon rings, which extend the π-conjugated systems. This modification not only narrows the bandgap but also accelerates the transport of photoelectrons and enhances the separation of electron–hole pairs, as illustrated in Figure 14. Owing to these synergistic effects, the H_2_ evolution rate of the in-plane carbon ring spliced multiple-ordered porous honeycomb structure of g-C_3_N_4_ (denoted as Cr–PHCN) has been largely increased, reaching 7581 mmol h^−1^ g^−1^, which is approximately 47.4 times higher than that of pristine CN (160 mmol h^−1^ g^−1^). Moreover, Cr–PHCN exhibits an impressive apparent quantum efficiency (AQE) of 10.62% at 420 nm, underscoring its exceptional photocatalytic performance.

Moreover, in pursuit of g-C_3_N_4_ with an expansive specific surface area and enhanced hydrogen evolution activity, our research has synthesized g-C_3_N_4_ nanosheets through the copolymerization of supramolecular self-assembled precursors with heterocyclic molecules. This process has resulted in a significant increase in both the specific surface area and carrier concentration, with the findings to be detailed in an upcoming publication. The experimental results indicate that the electronic structure of g-C_3_N_4_ was refined following copolymerization with heterocyclic molecules, and the specific surface area was further expanded upon the foundation of supramolecular self-assembly. Consequently, further investigation is warranted to elucidate the mechanisms behind the optimization of the electronic band structure and the doubling of the specific surface area by manipulating the nanoscale morphology and atomic-scale structures of g-C_3_N_4_. Additionally, a thorough evaluation of the hydrogen production performance and stability of the catalyst is essential to fully understand and leverage these enhancements.

## 6. Summary and Prospective

This review centers on g-C_3_N_4_, with a specific emphasis on addressing its inherent limitations. Beginning with a lot of previous research works, we find that the rational adjustment of the nanostructures and electronic/chemical structures of g-C_3_N_4_ can enhance the separation efficiency and density of photogenerated charge carriers. Significant and impactful advancements have been achieved in bolstering the photocatalytic H_2_ production capabilities of g-C_3_N_4_ under visible solar light, marking important strides in the field. Some of our own experiments have also been listed to embody the vital role of different strategies (element doping, atmosphere calcination, and copolymerization) on structural tailoring. The reasons for the formation of defects and their underlying mechanisms affecting the photocatalytic performance of g-C_3_N_4_ have been carefully discussed.

While g-C_3_N_4_ has been thoroughly investigated and has seen some exploratory applications in photocatalysis and various interdisciplinary domains, there remains a significant journey ahead to achieve our ultimate goal of sustainable development. Currently, there are still many bottlenecks to be overcome in improving the performance of these materials. Here, several new challenges and insights are presented: (1) The more detailed underlying mechanisms of charge carrier transport and recombination arising from the intrinsic molecular structure remain unclear. It seems that researchers are limited to typical carriers’ recombination and lifetime analysis, namely, an inhibited carrier recombination and extended fluorescence lifetime. (2) The current strategies mainly focus on the qualitative or semi-quantitative regulation of defects in g-C_3_N_4_-based catalysts. It is still difficult to undertake modifications of the surface/textural properties and bandgap configuration in a precise range. (3) Although the modulation of the electron band structure in g-C_3_N_4_ has yielded promising results, enhancing the absorption of visible light and even near-infrared light, it remains insufficient for achieving significant H_2_ production at longer wavelengths of light.

Consequently, there is a critical need for the rational design and development of innovative g-C_3_N_4_-based photocatalysts that can better address the demands of real-world usage. In this regard, we give some new insights according to the above discussion, as follows: (1) In addition to the widely recognized mechanisms of charge carrier transfer, a more comprehensive investigation into the thermodynamics and kinetics of surface catalytic reactions is also warranted. The adoption of novel characterization techniques and theoretical calculations could prove to be invaluable tools in this endeavor, potentially leading to a significant enhancement in our comprehension of the structure–activity relationship within g-C_3_N_4_-based photocatalysts. This will provide clearer theoretical guidance for the targeted design of new g-C_3_N_4_-based photocatalysts and expand the application fields of g-C_3_N_4_. (2) Given the numerous possibilities for substituting carbon with nitrogen in graphite in a regular pattern, we posit that the term ‘carbon nitrides’ encompasses a vast family of related compounds, such as C_3_N_4_, C_3_N_2_, C_3_N_5_, and so on. This opens up new avenues for an in-depth structural and nano-morphology control that could possess novel and potentially exciting properties. (3) Activation of n → π* electron transitions in amorphous carbon nitride may be an interesting strategy for long-wave light utilization. An in-depth exploration of amorphization may lead to some unexpected effects. The synergistic progress in defect design, characterization, and mechanism understanding is anticipated to pave the way for a new generation of defective g-C_3_N_4_-based photocatalysts. These advancements are expected to broaden their application spectrum, particularly in energy-related and environmental applications.

## Data Availability

Not applicable.
